# Specialty grand challenge editorial innovative approaches for pharmacoepidemiologic research in pregnancy: Shifting the paradigm of Thalidomide’s impact on pregnant women

**DOI:** 10.3389/fdsfr.2023.1187070

**Published:** 2023-03-31

**Authors:** Eva Gerbier, Alice Panchaud

**Affiliations:** ^1^ Service of Pharmacy, Lausanne University Hospital and University of Lausanne, Lausanne, Switzerland; ^2^ Materno-Fetal and Obstetrics Research Unit, Department “Woman-Mother-Child”, Lausanne University Hospital, Lausanne, Switzerland; ^3^ Institute of Primary Healthcare (BIHAM), University of Bern, Bern, Switzerland

**Keywords:** pregnancy, digital health (eHealth), pharmacoepidemiolgy, electronic databases, mobile application

## Introduction

Drug use during pregnancy is highly prevalent with 50%–81% of women reporting using at least one drug during that period (Mitchell et al., 2011; Lupattelli et al., 2014). These numbers are likely to keep increasing since women become pregnant at a later age and are more likely to have preexisting medical conditions and pregnancy complications. (Fridman et al., 2014). Even though drug use during pregnancy is commonplace, it has been estimated that nearly 98% of drugs approved by the Food and Drug Administration (FDA) between 2000 and 2010 carry an “undetermined” teratogenic risk (van Gelder et al., 2014). Furthermore, there is a dearth of information on how pregnancy-induced physiological changes impact a drug’s pharmacokinetics (Pinheiro and Stika, 2020). This uncertainty stems from the almost systematic exclusion of pregnant women from clinical trials following the thalidomide scandal.

The identification in 1961 of the first human teratogenic drug, thalidomide (i.e., treatment of nausea and vomiting in pregnancy), had a profound impact on biomedical research and drug regulation (Lenz, 1988). In 1971, the banning of diethylstillbestrol (i.e., medication used to prevent miscarriages) on the US market, after being associated with cervical and vaginal cancers in female offsprings, further enhanced the fear and suspicion generated by the thalidomide tragedy (Diethylstilbestrol DES Exposure and Cancer, 2021). As a result, the FDA issued a guideline in 1977 recommending the exclusion of most women of childbearing potential from early phases of clinical trials, a policy that was widely adopted by drug sponsors (Merkatz, 1998). It took more than 10 years for the FDA to identify the perverse impact of this policy on women’s health and to issue new guidelines (FDA, 2020a). These guidelines “Guideline for the Study and Evaluation of Gender Difference in the Clinical Evaluation of Drugs” removed the restriction on the inclusion of women in early phases of clinical trials and called for more studies on the pharmacokinetic differences between genders (FDA, 2020a).

Still, two decades later, clinical trials intended for pregnant women remain uncommon, with less than 0.5% of ongoing trials in 2013–2014 focusing on this group, and only 4% of those examining the pharmacokinetics of pregnancy (Scaffidi et al., 2017). Moreover, pregnancy and lactation are the most frequent exclusion criteria in clinical trials, as seen in a review of 38 new drugs approved by the FDA between 2014 and 2017 (Duggal et al., 2021). Consequently, much of what is known about medication safety during pregnancy is based on observational data gathered after the drugs are already on the market.

## Current methods for drug safety research in pregnancy

Observational data traditionally relied on data sources such as case reports, pharmacovigilance reports [spontaneous reporting system databases such as vigibase, case-control studies, and drug registries (ENTIS)]. While these methods have proved useful in identifying signals or confirming/invalidating potential risks, they also come with important limitations such as recall bias, selection bias, limited sample size because of time-consuming and costly recruitment, and potential loss to follow-up.

The substantial and constant increase in the digitalization of healthcare databases and the considerable progress in computer science have led to the emergence of an important data source for pharmacoepidemiologic research in pregnancy. These databases either include medical records or administrative databases (i.e., prescription records or insurance data). The United Kingdom Clinical Practice Research Datalink (CPRD) (Charlton et al., 2014), which comprises data routinely collected from medical and prescribing records, is one such example. It has been used to evaluate many drug-related outcomes during pregnancy such as the risk of congenital anomalies linked to first trimester exposure to antidepressants [tricyclics and selective serotonin reuptake inhibitors (SSRIs)] (BCDSP, 2022) or to antihypertensives (Vasilakis-Scaramozza et al., 2013). Another example of electronic database analysis is the Medicaid Analytic eXtract (MAX) insurance data (Palmsten et al., 2013). The association between several drug exposures during early pregnancy and major malformations overall or medication use patterns were assessed using this database (Brogly et al., 2018). One limitation of these electronic databases is often the absence of important variables on exposure and confounders, which may not be present as it was not collected with the study’s aims in mind. Furthermore, since the beginning of pregnancy is often not recorded in these databases or imprecisely, gestational age needs to be estimated based on pregnancy exams or delivery codes.

This may be counteracted by the linkage of multiple databases based on a unique identifier, which is the case in several Nordic countries (Denmark, Finland, Iceland, Norway, Sweden) (Kieler, 2014). These countries have set up birth registers in the 1970s (Kieler, 2014). Unfortunately, it is only about 20 years later that they started recording information on drug use. These birth registries can be linked to other health registers (i.e., cancer register, patient register, cause of death register, and disease specific registers) offering a unique wealth of information. One example is the use of the Danish National Birth Cohort (DNBC) to assess the relation between several exposures and outcomes such as prenatal antidepressant use and child behavioral outcomes at 7 years of age (Grzeskowiak et al., 2016).

Yet, combining data from multiple registers can be a time-consuming process, and when coupled with delays in updating certain registers, obtaining the most current information may not always be feasible. Furthermore, this is not feasible in all countries due to legal and political restrictions over data privacy [ref].

## Future perspectives

### Fair inclusion of pregnant women in clinical trials

Recently, many researchers and organizations have emphasized the need for creating frameworks that ensure fair inclusion of pregnant women in clinical trials (Lyerly et al., 2008; White, 2015). For instance, in 2018 a list of recommendations on research specific to pregnant women and lactating women (PRGLAC) was published by the Task Force on Research Specific to Pregnant Women and Lactating Women (PRGLAC, 2019). In parallel, the FDA published a guidance on how to include pregnant women in clinical trials (FDA, 2020b).

The COVID-19 pandemic presented an excellent opportunity to promote their inclusion since most of the tested therapies were repurposed medicines that had been previously administered to pregnant women, such as chloroquine (Chloroquine, 2020), the lopinavir-ritonavir combination (Clinicalinfo, 2023), or remdesivir (Mulangu et al., 2019). Moreover, based on past observations of the impact of other respiratory viruses (Valentine et al., 2020), there was a significant suspicion of potential risk posed by COVID-19 to pregnant mothers and their fetuses. Consequently, several initiatives called for the participation of women in COVID-19 clinical trials as illustrated by a letter sent to the FDA from the Coalition to Advance Maternal Therapeutics (CAMT), which included the American Academy of Pediatrics, the American College of Obstetricians and Gynecologists, the Society for Maternal-Fetal Medicine, or the Organisation of Teratology Information Services (OTIS), amongst others (Francis and Stephen, 2020). Still, in April 2020, pregnancy was listed as an exclusion criterion in 74% of COVID-19 clinical trials evaluating repurposed drugs with no or low safety concerns during pregnancy (Taylor et al., 2021).

Not only pregnant women were excluded from treatment clinical trials but to an even greater extent from COVID-19 vaccine trials, with 98.7% (88/90) of trials excluding pregnant women between May and October 2020 (Taylor et al., 2021). It is only once data on the vaccines’ safety had appeared among women who unintentionally became pregnant during the trials, that vaccines were evaluated on pregnant women during clinical trials (Pfizer, 2023). This delayed inclusion had dire consequences in the perinatal population. Indeed, some pregnant women were thus denied the possibility of protecting themselves and their future babies from severe, and even deadly, outcomes (Villar et al., 2021). The absence of adequate high-quality data and consensus regarding their inclusion in the trials likely caused fear and a lack of trust among those who were subsequently given the chance to get vaccinated (Carbone et al., 2022). Still, vaccination rates among pregnant women remain lower than in the general population (Stock et al., 2022).

After the missed opportunity to include pregnant women in COVID-19 treatment and vaccine trials, it seems obvious that fair inclusion of pregnant women in clinical trials is not yet for tomorrow.

Thus, finding new approaches to collecting data in order to foster research on drug safety during pregnancy is still on the agenda. In this regard, digital health platforms such as social media and pregnancy applications could offer promising avenues for conducting research in this population.

### Digital health

In 2022, it was estimated that 59% of the world’s population used at least one type of social media, with Facebook, Youtube, and Instagram being the most popular (Smart Insights, 2022). This proportion is even higher among the young adults, with an estimated 90% of American adults aged between 18 and 29 using social media in 2016 (SHANNON et al., 2016). However, only recently have researchers tried to exploit the widespread use of social media as a new tool to conduct research.

#### Social media

One way this has been done in pregnancy pharmacoepidemiology was to establish pregnancy cohorts directly based on the content of social media (Sarker et al., 2017). As an example, in 2017, twitter postings (tweets) were analysed using natural language processing to determine whether a woman was pregnant and automatically retrieve information on her pregnancy, including drug use (Sarker et al., 2017). The same authors also used twitter posts in 2018 to create a cohort of pregnancies with birth defect outcomes (Klein et al., 2018), in which they found congenital heart defects to be the most common congenital anomaly reported, similarly to reports using standard methods. Among the advantages of this method, the authors highlighted the availability of data in both the pre-conceptional period and *postpartum* for most twitter users. This method is probably at risk of underreporting since not all pregnant women use twitter and not all twitter users will post about their pregnancy or its outcome. The European Union’s Innovative Medicines Initiative advises against using social media for general monitoring of adverse effects, but recognizes its usefulness in specific areas such as drug abuse and pregnancy-related outcomes. They also acknowledge that further research could lead to an expansion of its scope and utility (van Stekelenborg et al., 2019).

In parallel, a large proportion of pregnant women use a pregnancy mobile application during that period with estimates varying between 55% in the United States to 75% in Australia (Lupton and Pedersen, 2016; Frid et al., 2021). Until now, researchers mostly perceived mobile applications as a mean to promote study recruitment (Vignato et al., 2019) or healthy behaviors during pregnancy such as controlling gestational weight gain (Halili et al., 2018), promoting physical exercise (Chan and Chen, 2019) and improving mental health (Evans et al., 2022).

#### Pregnancy mobile applications

A few initiatives have been carried out to use data collected on pregnancy mobile applications to directly study drug utilization and safety. For example, the Rhekiss mobile application was developed as a complementary tool to the already existing web-based app to fulfil data for the German pregnancy register Rhekiss (Richter et al., 2021). The implementation of the application allowed a slight increase in the proportions of submitted forms (i.e. 5%). More interestingly is that authors observed that patients using the application rather than the website tended to be younger and less educated. In 2017, as part of the FDA MyStudies app, pregnant women were recruited through Kaiser Permanente Washington, an integrated healthcare delivery system in the US (Rothschild et al., 2022). After downloading the application and completing an app-based consent form, they were asked to complete baseline questionnaires directly on the app. Additional questionnaires were sent during a 3-month follow-up period. The authors compared data obtained through the app to the one recorded in the electronic health record (EHR). They observed that the application had the potential to obtain information on over-the-counter drugs and sensitive behaviors such as alcohol and illicit drug use, which is often missing or incomplete in EHRs. They also noted its utility to record self-reported discontinuation of prescription drugs which may be helpful to reduce exposure misclassification in certain drug classes (e.g., psychotropics). In this study, participants were older than the rest of pregnant women in the EHR and had higher outpatient healthcare utilisation, perhaps suggesting that women who are more invested in the healthcare system may be more willing to enroll. Finally, in 2021, to evaluate the use of prophylactic low dose aspirin (LDASA) in pre-eclampsia, self-reported use of LDASA in MyHealthyPregnancy application was compared to recommendations of prophylactic LDASA in the medical records (Krishnamurti et al., 2021). Furthermore, authors assessed risk factors for pre-eclampsia which were most associated with a recommendation of LDASA. Cross examination of medical records and the application showed that a recommendation had been given to almost 70% of patients with high-risk criteria while less than half of patients reported receiving a recommendation for LDASA in the application. Thus, this study highlighted the potential for a pregnancy application to improve identification of patients who would benefit from prophylactic LDASA and gaps in patient-prescriber communication.

Interestingly, all the examples reported here and found in the literature until today are that of apps that collect data only on certain aspects of pregnancy and that do not provide their users with benefits in exchange. However, to tackle the recurrent problem of confounders, these apps should inquire about other aspects than medication use and obstetric outcomes alone, such as nutrition, physical activity, alcohol/drug consumption. This type of data is very rarely available in other data sources. Furthermore, to promote sufficient study recruitment and women’s engagement in providing data, these apps could incorporate certain features that women find desirable. For instance, health information, obtained immediately at minimal to no cost, has been highlighted as one of the most valued functionalities of these apps (Sommer et al., 2017). However, the absence of regulation of the app’s content can lead to non-evidence based, poor quality information, which can in turn be harmful to their users. This was highlighted in a 2017 review assessing the nutritional content of 51 pregnancy applications in Australia (Wiley, 2022). Similar findings were observed in a United Kingdom review of 29 apps in which authors found some of the information provided to be potentially harmful to pregnant women (Catherine et al., 2020).

To conclude, each data source has its strengths and limitations in drug safety research in pregnancy. If pooling data sources would improve the completeness of the available information, it would still not provide the ideal dataset with all information needed. Thus, there is a need for the development of new approaches. Social media and pregnancy mobile applications to collect data on the perinatal population is one of the possible avenues. It is still in its early stages and future work should be carried out to prove the potential of these tools.

With this new specialty section on maternal and fetal medicine, we hope to report paper reflecting the transition of research to “protect mothers and infant through research instead of from research” as wisely declared by NICHD Director Diana W. Bianchi, M.D.
